# MR-proADM Predicts Mortality and Heart Failure Events in ATTR Cardiac Amyloidosis

**DOI:** 10.1161/CIRCULATIONAHA.125.077833

**Published:** 2026-03-31

**Authors:** Belén Peiró-Aventín, Elena Revuelta-Lopez, Mariana Brandão, Julio Nuñez, Manuel A. Fernández-Rojo, Rosa M. Carmona, Borja Montejo, Alejandro Ferrando-Muñoz, Aránzazu Martín-García, Esther Gonzalez-Lopez, Fernando Dominguez, Andrea Camblor, María Ruiz-Cueto, Josep Lupon, Sergio Teruya, Dimitrios Bampatsias, Israt Jahan, Ana Royuela, Ahmad Masri, Mathew S. Maurer, Antoni Bayes-Genis, Pablo García-Pavía

**Affiliations:** 1 Department of Cardiology, Hospital Universitario Puerta de Hierro Majadahonda, IDIPHISA, Majadahonda, Spain (B.P.-A., M.B., E.G.-L., F.D., P.G.-P.).; 2 Centro Nacional de Investigaciones Cardiovasculares (CNIC), Madrid, Spain (B.P.-A., M.A.F.-R., R.M.C., F.D., P.G.-P.).; 3 Cardiology Department, Hospital Universitari Germans Trias i Pujol, Badalona, Department of Medicine, Autonomous University of Barcelona, Barcelona, Spain (E.R.-L., A.C., M.R.-C., J.L., A.B.-G.).; 4 CIBERCV, Instituto de Salud Carlos III, Madrid, Spain (E.R.-L., J.N., E.G.-L., F.D., M.R.-C., J.L., A.B.-G., P.G.-P.).; 5 ICREC Research Program, Germans Trias i Pujol Research Institute (IGTP), Badalona, Barcelona, Spain (E.R.-L., B.M., A.B.-G.).; 6 Cardiology Department, Hospital Clínico Universitario de Valencia, Universitat de Valencia, INCLIVA, Valencia, Spain (J.N.).; 7 The University of Queensland, Frazer Institute, Brisbane, Australia (M.A.F.-R.).; 8 Department of Biochemistry, Hospital Universitario Puerta de Hierro Majadahonda, Majadahonda, Spain (A.F.-M., A.M.-G., A.J.-M.).; 9 Clinical Cardiovascular Research Laboratory for the Elderly (CCRLE), New York-Presbyterian/Columbia University Irving Medical Center, New York, NY (S.T., D.B., M.S.M.).; 10 Cardiovascular Institute, Oregon Health and Science University, Portland, OR (I.J., A.M.).; 11 Clinical Biostatistics, Puerta de Hierro Biomedical Research Institute (IDIPHISA), CIBERESP, Madrid, Spain (A.R.).; 12 Universidad Francisco de Vitoria (UFV), Pozuelo de Alarcón, Spain (P.G.-P.).

**Keywords:** biomarkers, MR-proADM, prognosis, transthyretin

## Abstract

**BACKGROUND:**

With the increasing diagnosis of transthyretin amyloid cardiomyopathy (ATTR-CM) at earlier stages and new therapies, there is a rising demand for tools to stratify risk and prognosis. We evaluated the prognostic value of multiple circulating biomarkers for predicting outcomes in ATTR-CM.

**METHODS:**

We evaluated 12 different circulating biomarkers (N-terminal pro-B-type natriuretic peptide [NT-proBNP], high-sensitivity troponin I [hsTnI], mid-regional pro-adrenomedullin [MR-proADM], carbohydrate antigen 125 [CA125], soluble suppression of tumorigenicity 2 [sST2], cluster of differentiation antigen 146 [CD146], growth/differentiation factor-15 [GDF-15], alpha-klotho, fibroblast growth factor 23 [FGF-23], galectin-3, insulin-like growth factor-binding protein 7 [IGFBP-7], and estimated glomerular filtration rate [eGFR]) in 337 ATTR-CM patients from Spain. Cox models were employed to determine their predictive abilities. Findings were validated in 2 independent external cohorts of 210 patients from the United States and 416 patients from the ATTR-ACT trial, respectively.

**RESULTS:**

Over a median follow-up of 19.7 months (IQR, 6.5–42.3), 67 patients (19.9%) died/underwent heart transplantation, and 81 (24%) had heart failure events. MR-proADM was the biomarker with the strongest prognostic performance, with a C-index of 0.788 (95% CI, 0.723–0.851) for all-cause mortality and 0.721 (95% CI, 0.669–0.772) for the composite end point of death and heart failure events. MR-proADM was associated with multiple parameters of ATTR-CM severity and was independently associated with mortality, heart failure events, and the composite end point. MR-proADM ≥1.1 nmol/L was identified as the optimal prognostic threshold, and it improved prediction of mortality when added to the National Amyloid Center (area under the curve [AUC], 0.682 versus 0.737; *P*<0.001), Mayo (AUC, 0.659 versus 0.749; *P*<0.001), and the Columbia staging systems (AUC, 0.699 versus 0.768; *P*<0.001). In both validation cohorts, patients with MR-proADM ≥1.1 nmol/L had worse outcomes (*P*<0.001). This association was also confirmed in patients receiving tafamidis.

**CONCLUSIONS:**

In patients with ATTR-CM, MR-proADM levels are associated with disease severity and worse prognosis. MR-proADM improves prediction of all-cause mortality and captures heart failure events.

**Clinical Perspective**

**What Is New?**

- Circulating mid-regional pro-adrenomedullin (MR-proADM) is associated with markers of heart failure and disease severity in patients with transthyretin amyloid cardiomyopathy.
- Mid-regional pro-adrenomedullin predicts mortality and heart failure events in transthyretin amyloid cardiomyopathy, even after adjustment for established prognostic factors such as NT-proBNP (N-terminal pro-B-type natriuretic peptide), estimated glomerular filtration rate, diuretic dose, and New York Heart Association functional class.
- Mid-regional pro-adrenomedullin ≥1.1 nmol/L identifies patients at high risk of events and improves mortality prediction when added to the Mayo, National Amyloid Center, and Columbia staging systems.

**What Are the Clinical Implications?**

- Incorporating mid-regional pro-adrenomedullin into routine clinical testing and established staging systems could improve risk stratification and guide clinicians in selecting the most appropriate therapeutic strategy in patients with transthyretin amyloid cardiomyopathy.

Transthyretin amyloid cardiomyopathy (ATTR-CM) is a progressive condition produced by the deposition of misfolded transthyretin (TTR) monomers, forming amyloid fibrils.^1,2^ This process leads to the development of a restrictive cardiomyopathy, frequently associated with poor long-term prognosis.^3^ The disease can stem from destabilizing variants in the *TTR* gene (ATTRv) or can occur in individuals with wild-type TTR in a process associated with aging (ATTRwt).^2^

Over the past decade, significant advances have been made in understanding the disease. While its real prevalence remains unknown, what was once considered a rare condition, predominantly affecting elderly men and usually diagnosed at an advanced phase, is now increasingly identified at earlier stages because of advances in noninvasive diagnosis and the availability of disease-modifying treatments.^4^ Moreover, ATTR-CM is now frequently recognized among patients with several common clinical scenarios such as heart failure with preserved ejection fraction, severe aortic stenosis, and hypertrophic cardiomyopathy.^4–6^

As more patients with ATTR-CM are diagnosed at earlier stages of the disease and have access to new specific treatments, there is an increasing need for specific tools to stratify their risk and prognosis accurately.

Classic biomarkers such as N-terminal pro-B-type natriuretic peptide (NT-proBNP), estimated glomerular filtration rate (eGFR), and troponin are established predictors of mortality in ATTR-CM patients, becoming key components of current prognostic scores.^7,8^ However, these scores primarily focus on survival and were developed years ago, when patients were frequently diagnosed at advanced disease stages. With the increasing life expectancy of ATTR-CM patients today, there is a growing interest in assessing not only mortality but also in identifying individuals at risk of heart failure decompensation. Although there are no staging systems to predict heart failure related events in ATTR-CM, heart failure episodes are associated with worse prognosis^9^ and are frequently included in the primary end points of the clinical trials assessing new drugs to treat this disease.

Accordingly, we sought to evaluate the prognostic role of 12 circulating biomarkers previously described in heart failure as predictors of all-cause mortality and heart failure events in patients with ATTR-CM.

## Methods

The data that support the findings of this study are available from the corresponding author upon reasonable request. Qualified researchers may submit a request containing the research objectives, end points/outcomes of interest, a statistical analysis plan, data requirements, a publication plan, and qualifications of the researcher(s). Requests will be reviewed by corresponding authors. If approved, information necessary to address the research question will be provided under the terms of a data sharing agreement. Requests may be submitted to corresponding authors.

### Participants

Consecutive patients diagnosed with ATTR-CM from Hospital Universitario Puerta de Hierro Majadahonda (Madrid, Spain) and Hospital Universitari Germans Trias I Pujol (Badalona, Barcelona, Spain) who had a blood sample collected were included. Baseline assessments were performed between December 2014 and October 2022, with the majority of patients (99.1%) enrolled from January 2017 onward. The study was approved by the Hospital Universitario Puerta de Hierro Majadahonda ethics committee and in agreement with the principles of the Helsinki Declaration. Patients provided written consent for collection of samples.

ATTR-CM was diagnosed invasively and noninvasively by: (1) demonstration of TTR amyloid deposits on endomyocardial biopsy; (2) demonstration of TTR amyloid deposits on extracardiac biopsy and echocardiographic or cardiac magnetic resonance (CMR) criteria (ie, diffuse subendocardial or transmural late gadolinium enhancement with abnormal gadolinium kinetics); and (3) cardiac uptake grade 2 or 3 on 3,3-diphosphono-1,2-propanodicarboxylic acid single photon emission computed tomography (DPD SPECT) scintigraphy and no evidence of monoclonal protein (negative serum and urine immunofixation and negative serum free light chains) in the presence of echocardiographic or CMR criteria for cardiac amyloidosis according to the European Society of Cardiology position paper on diagnosis and treatment of cardiac amyloidosis.^1^ Both patients with ATTRv and ATTRwt were enrolled.

Results were validated in 2 independent external cohorts. The first validation cohort included 210 patients with ATTR-CM from 2 centers from the United States: Oregon Health and Science University Medical Center (Portland, OR), and Columbia University Irving Medical Center (New York, NY). The same inclusion criteria for the derivation cohort were applied. The second validation cohort included 416 patients from the ATTR-ACT trial, a phase 3, multicenter, placebo-controlled, double-blind, randomized trial that evaluated the safety and efficacy of tafamidis in ATTR-CM.^10^ ATTR-ACT trial included ATTR-CM patients with both hereditary and wild-type ATTR-CM; main inclusion criteria comprised an age between 18 and 90 years, New York Heart Association (NYHA) class I to III, and a baseline NT-proBNP ≥600 pg/mL. Participants were assigned in a 2:1:2 ratio to receive 80 mg of tafamidis, 20 mg of tafamidis, or placebo, respectively. Out of the 441 subjects randomized in the ATTR-ACT trial, 416 had a baseline blood sample available and were included in this analysis.

### Baseline Clinical Characteristics

Demographic and clinical data at baseline evaluation were collected from clinical records in the initial and the first validation cohort using a uniform methodology. Age, type of ATTR-CM, NYHA class, and medical therapy including ATTR specific therapies were obtained. Presence of comorbidities such as ischemic cardiomyopathy or previous stroke or atrial fibrillation were also collected. Baseline characteristics included eGFR, calculated using the CKD-EPI (Chronic Kidney Disease Epidemiology Collaboration) formula, cardiac biomarkers, and echocardiographic parameters obtained at first evaluation. ECG findings were also collected. Torasemide doses were converted to furosemide equivalents, using a ratio of 20 mg of torasemide to 40 mg of furosemide to standardize furosemide dosing. Baseline clinical characteristics of patients from ATTR-ACT were obtained from eCRF.

### Outcomes

The study outcomes included all-cause mortality, worsening heart failure (defined as hospital admission for heart failure or ambulatory administration of intravenous diuretics to treat congestive symptoms), and a composite end point of all-cause mortality, worsening heart failure, or heart transplant. Events were collected by investigators blinded to biomarker levels through a thorough review of medical records and by contacting patients to evaluate their survival status. Outcomes of patients from the ATTR-ACT cohort were captured from the study database. Patients were followed until death or last available clinical contact, with October 2023 being the last censoring date. For survival analyses, follow-up was truncated at 3 years, such that patients with longer follow-up were censored at that time point.

### Biomarkers

Carbohydrate antigen 125 (CA125), insulin-like growth factor-binding protein 7 (IGFBP7), galectin-3, cluster of differentiation antigen 146 (CD146), growth/differentiation factor-15 (GDF-15), alpha-klotho, soluble suppression of tumorigenicity biomarker 2 (sST2), mid-regional pro-adrenomedullin (MR-proADM) and fibroblast growth factor 23 (FGF23) and high-sensitivity troponin I (hsTnI) levels were determined in a blinded manner at Centro Nacional Investigaciones Cardiovasculares (CNIC; Madrid, Spain), at Hospital Universitario Puerta de Hierro (Madrid, Spain) or at Hospital Universitari Germans Trias I Pujol (Barcelona, Spain) by immunoassays in EDTA-plasma and serum/plasma samples obtained from patients with ATTR-CM from the derivation cohort. Additionally, NT-proBNP and eGFR were collected from baseline evaluation at participating centers. Biomarkers examined were selected based on their previous description as prognostic factors in cohorts of patients with heart failure and the availability of commercial immunoassays.^11,12^ The analysis method followed for each biomarker was according to the manual provided by the manufacturer. A more detailed description of the methods used to measure each biomarker is available in the Supplemental Material.

Subsequently, MR-proADM levels were measured in a blinded manner in plasma samples from patients from the 2 validation cohorts following the same methodology as in the derivation cohort.

### Statistical Analysis

Baseline continuous and categorical variables are presented as median (interquartile range) and frequencies (percentage), respectively. Survival estimations were evaluated from the first medical contact to the last follow-up or death. Follow-up of patients enrolled in double-blind, placebo-controlled clinical trials in the derivation and the first validation cohorts was censored at the time of trial enrollment.

Initially, a univariable Cox regression model was employed to assess the predictive performance of the 12 biomarkers for all-cause mortality and the composite end point. The biomarker with strongest predictive capacity was selected for further analysis. To evaluate the performance of MR-proADM over time, follow-up was censored at 3 years for all survival-time analyses. This time frame was selected based on previous studies^13^ because of the reported median survival of patients with ATTR-CM^14^ and because of the high rate of early events in these patients, making models less stable over extended periods.

Subsequently, we evaluated MR-proADM according to clinical, ECG, and echocardiographic parameters to evaluate its association with markers of disease severity. Patients were stratified based on MR-proADM levels, using the established standard cutoff. Characteristics and outcomes were compared between groups using the Mann-Whitney *U* test for continuous variables and the Pearson χ^2^ test for categorical variables.

Given the potential nonlinear relationship between levels of biomarkers and the risk of events, MR-proADM risk gradient across all its values was assessed using fractional polynomial regression after univariable and multivariable approaches, with model selection based on Akaike and Bayesian information criteria. The best fitting model was a first-order fractional polynomial with a power of −1.0. The first multivariable model included NT-proBNP and eGFR (variables included in the UK National Amyloid Center [NAC] prognostic staging system^7^), whereas the second model incorporated NT-proBNP, eGFR, diuretic dose indexed by body weight, and NYHA functional class (variables included in the Columbia prognostic staging system^13^).

The optimal cutoff for MR-proADM was selected using time-dependent receiver operating curve analysis with nearest-neighbor estimation based on the method proposed by Heagerty, Lumley, and Pepe,^15^ with the cutoff selected using the Perkins and Schisterman criterion (stroccurve instruction in STATA).^16,17^ The cutoff was estimated using 1-year mortality, as this time frame minimizes biomarker variability over longer follow-up periods and provides a reliable association between MR-proADM levels and outcomes. Kaplan-Meier curves were plotted for the composite end point and all-cause mortality. For worsening heart failure, cumulative incidence curves were estimated using a competing risk regression model based on the Fine and Gray method,^18^ with death considered as the competing event.

The incremental value of MR-proADM combined with the NAC and the Columbia staging systems was evaluated by comparing the predictive performance of the survival models using Harrell’s C-statistic and by assessing their areas under the curve (AUC) for predicting 3-year mortality. We also evaluated the incremental value of MR-proADM to the Mayo staging system. Since troponin T was not available, hsTnI was determined instead. Accordingly, we applied the cutoff of 80 ng/L, proposed by De Michieli et al,^19^ hereafter referring to this approach as a modified Mayo staging system.

Finally, the results were validated using 2 external validation cohorts. Although survival was assessed in all patients, worsening heart failure events were not fully captured in a large proportion of patients from the first validation cohort (US cohort) because many individuals were primarily followed at local centers. Therefore, this cohort was used exclusively to validate the results for all-cause mortality. The second cohort (ATTR-ACT cohort), obtained from a clinical trial setting, provided data on mortality and heart failure hospitalizations, allowing validation of both all-cause mortality and the composite end point.

All statistical tests were 2-sided. A value of *P*<0.05 was considered statistically significant. Analyses were performed using STATA (version 16; StataCorp).

## Results

### Baseline Characteristics and Outcomes

A total of 337 ATTR-CM patients comprised the initial cohort, 296 (87.8%) from Hospital Universitario Puerta de Hierro and 41 (12.2%) from Hospital Universitari Germans Trias I Pujol. The median time from definitive ATTR-CM diagnosis to blood sample collection was 62 days (IQR, −27 to 263 days), and the median follow-up time was 19.7 months (IQR, 6.5–42.3 months).

The median age of the overall cohort was 78.3 years (73.1 to 82.9), 276 (81.9%) were men, 291 (86.35%) had ATTRwt, and 92 (27.3%) were receiving tafamidis at baseline or initiated it during the initial 12 months of follow-up. Among the 46 patients with ATTRv, the most common variant was Val142Ile (16 patients). Most patients were in NYHA class II (182 patients; 54%), whereas 92 (27.3%) were in NYHA I, and 63 (18.7%) were in NYHA III. Median eGFR was 61 mL/min (47–78 mL/min) and NT-proBNP 2209 pg/mL (942–4140 pg/mL). Two thirds (228; 67.7%) of the patients were under diuretic treatment, and the median furosemide dose was 0.47 mg/kg (0–0.87 mg/kg). Additional baseline characteristics are presented in Table 1.

**Table 1.**
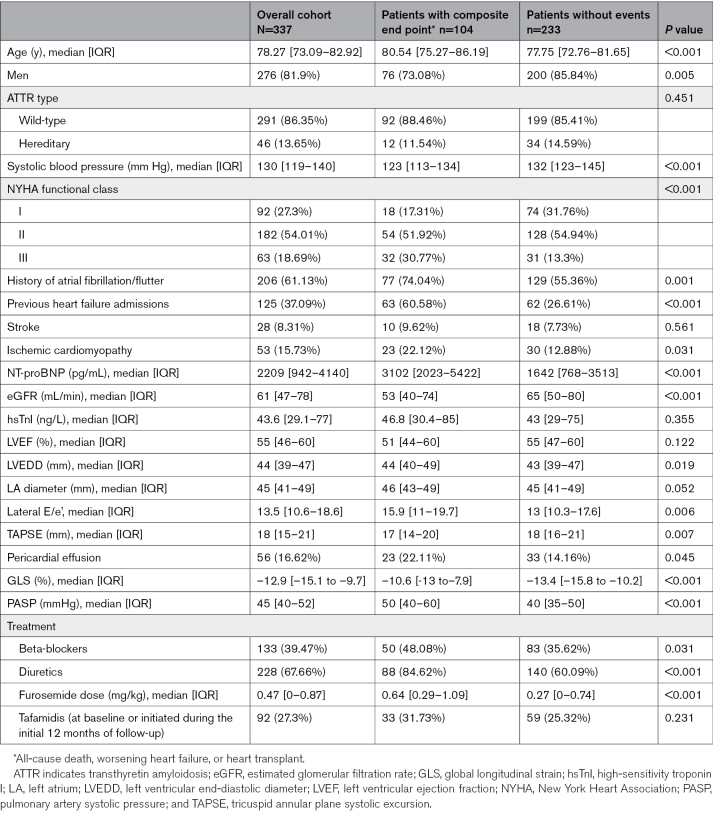
Baseline Characteristics

According to the NAC staging system, 187 patients (55.5%) were classified as stage I, 98 (29.1%) as stage II, and 52 (15.4%) as stage III. Median survival by stage was 74.5 months (95% CI, 74.4; could not be estimated) for stage I, 44.2 months (95% CI, 32.1; could not be estimated) for stage II, and 36.6 months (95% CI, 23.2; could not be estimated) for stage III.

Throughout the follow-up period, 66 (19.6%) patients died, 81 (24%) experienced heart failure events, and one (0.3%) underwent heart transplant. A total of 80 (23.7%) patients were censored because of inclusion in a double-blind, placebo-controlled clinical trial, with a median follow-up time before enrollment in the clinical trial of 123 [91–203] days. The median survival time of the overall cohort was 6.2 years (95% CI, 5.5 years; could not be estimated). The estimated 1-year survival was 93% (95% CI, 89.2–95.5), 3-year survival was 74.5% (95% CI, 67.5–80.2), and 5-year survival was 62.8% (95% CI, 53.9–70.5). Kaplan Meier curves for mortality, worsening heart failure, and the composite end point are displayed in Figure S1 from Supplemental Material.

As shown in Table 1, patients with events were more likely to be in a more advanced NYHA class, have a history of atrial fibrillation/flutter (AF/AFL), previous heart failure admissions, elevated NT-proBNP levels, and reduced eGFR values. They were more commonly treated with diuretics and beta-blockers and presented more frequently with echocardiographic findings of advanced disease, such as lower tricuspid annular plane systolic excursion (TAPSE), higher lateral E/e’, pulmonary artery systolic pressures (PASPs), and global longitudinal strain (GLS). Pericardial effusion was also more frequent in these patients.

We performed site-stratified analyses comparing patients from Hospital Universitario Puerta de Hierro Majadahonda (N=296) and Hospital Universitari Germans Trias i Pujol (N=41). Patients from Puerta de Hierro were younger, more often men, and more frequently in advanced NYHA class, with a higher prevalence of ATTRv and tafamidis use, as expected in a national referral center. Although minor echocardiographic and analytical differences were also noted, there was no evidence that patients from either hospital were systematically in a more advanced disease stage (Table S1).

#### Prognostic Role of Circulating Biomarkers

The prognostic performance of circulating biomarkers was individually assessed in 2 regression models to predict all-cause death and the composite end point. Harrell’s C-index values were calculated to assess the predictive capacity of the biomarker (Table 2). MR-proADM demonstrated the strongest prognostic performance, with a C-index of 0.788 (95% CI, 0.723–0.851) for all-cause mortality and 0.721 (95% CI, 0.669–0.772) for the composite end point. Based on these results, MR-proADM was selected for further analysis.

**Table 2.**
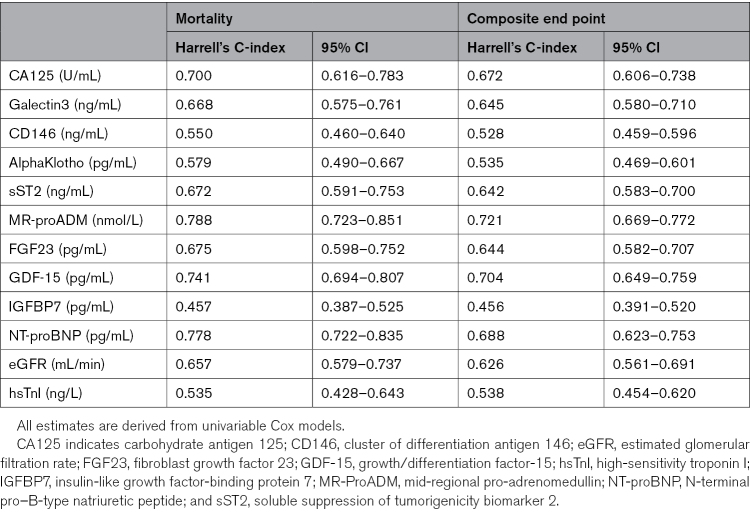
Prognostic Performance of Biomarkers for Prediction of All-Cause Mortality and the Composite End Point.

#### Association of MR-proADM With Markers of Severity

Median baseline level of MR-proADM was 0.816 (0.617–1.12) nmol/L. A total of 174 patients (51.6%) had concentrations of MR-proADM >0.79 nmol/L, which is the cutoff value proposed in chronic heart failure.^20,21^

Patients with MR-proADM measurements above this cutoff value exhibited more frequently several markers of disease severity (Table 3). These included a more advanced NYHA functional class, history of AF, previous admissions for heart failure, lower left ventricular ejection fraction (LVEF), parameters of diastolic dysfunction, presence of pericardial effusion, elevated PASP and GLS, reduced TAPSE, and higher doses of loop diuretics. Moreover, patients with higher MR-proADM also exhibited higher NT-proBNP and lower eGFR.

**Table 3.**
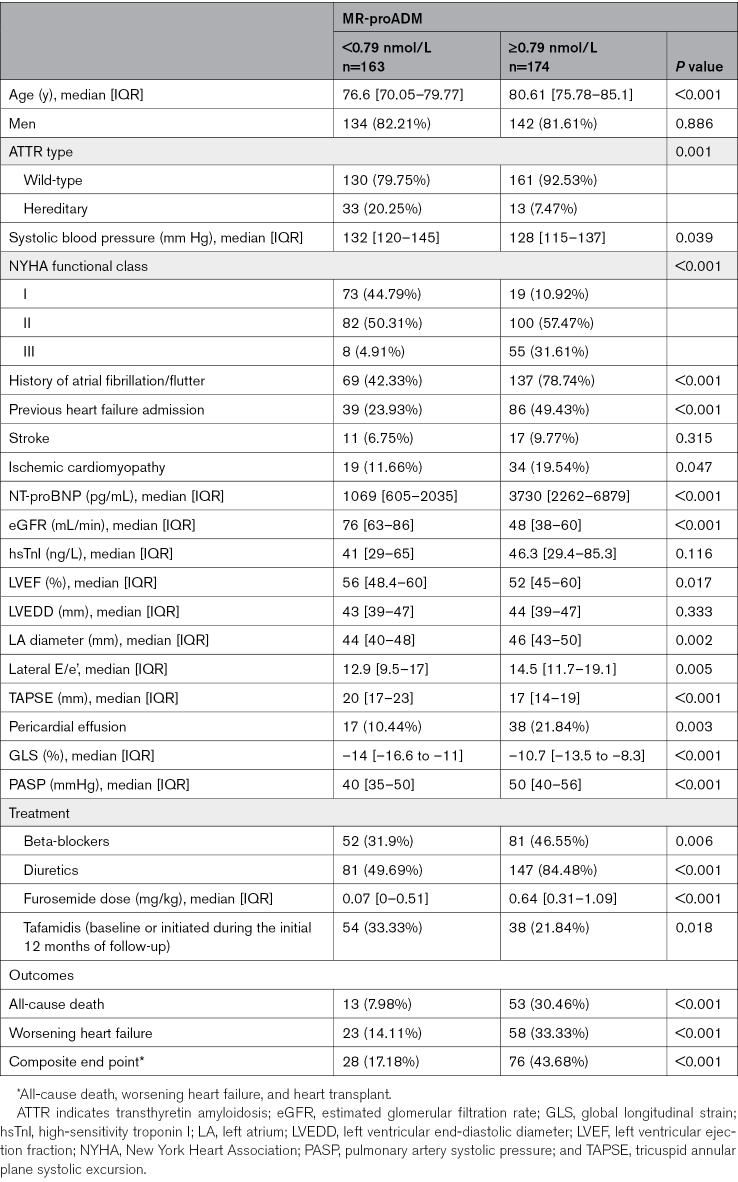
Association of MR-proADM With Severity of Disease

#### Association of MR-proADM Levels With Prognosis

**Figure 1.**
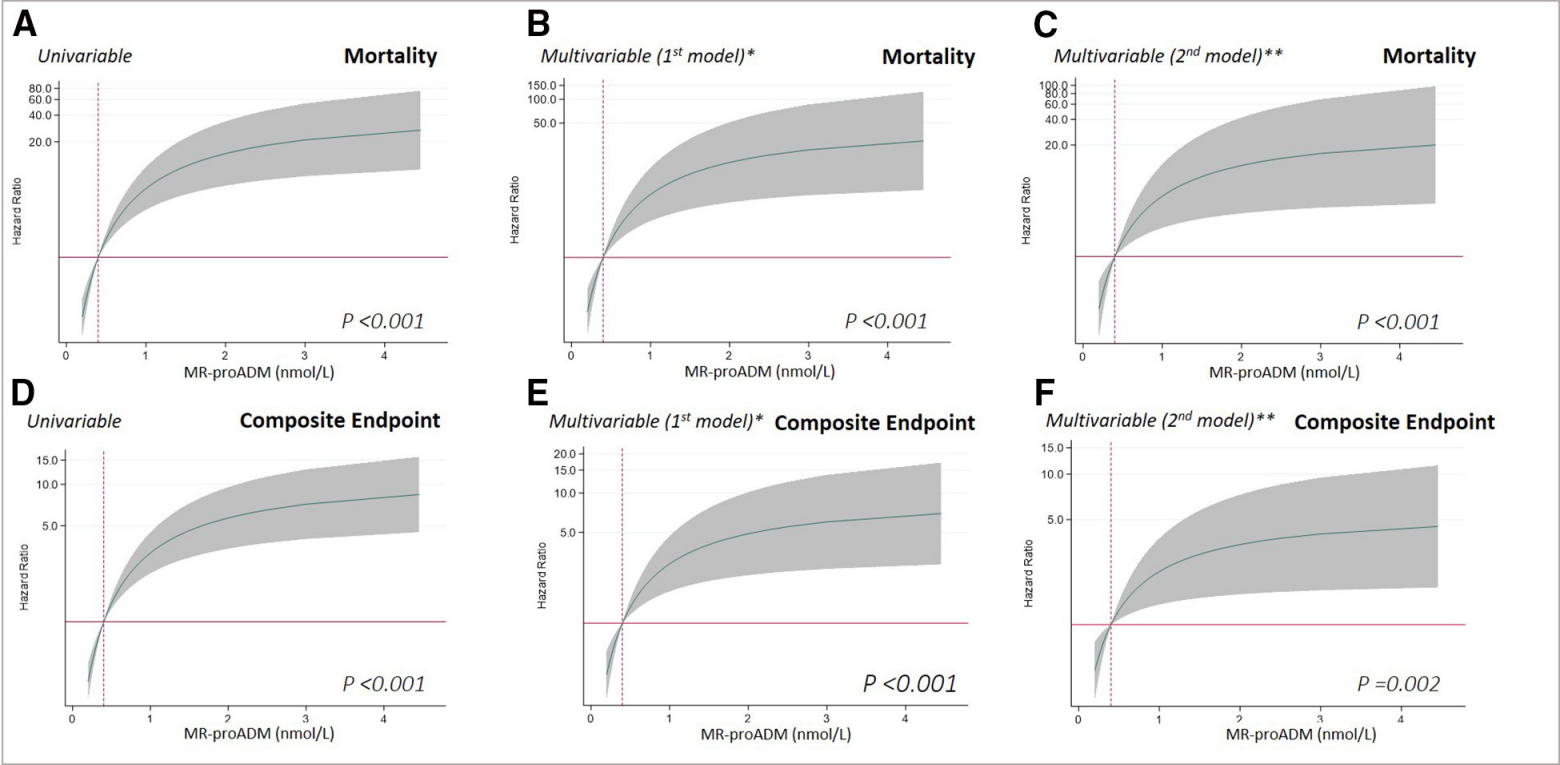
**Gradient of risk of MR-proADM levels for predicting all-cause death and the composite end point in patients with ATTR-CM.** Multivariable model 1 included MR-proADM, NT-proBNP, and estimated glomerular filtration rate (eGFR). Multivariable model 2 included MR-proADM, NT-proBNP, eGFR, New York Heart Association functional class, and diuretic dose (indexed). Hazard ratios were estimated using fractional polynomial regression; shaded areas represent 95% CIs. **A** through **C**, Association between MR-proADM levels and all-cause death in the univariable model (**A**), multivariable model 1 (**B**), and multivariable model 2 (**C**). **D** through **F**, Association between MR-proADM levels and the composite end point in the univariable model (**D**), multivariable model 1 (**E**), and multivariable model 2 (**F**). MR-proADM indicates mid-regional pro-adrenomedullin; and NT-proBNP, N-terminal pro-B-type natriuretic peptide.

Patients with MR-proADM above the normal cutoff values had higher mortality, more worsening heart failure events and higher incidence of the composite end point than patients who had MR-proADM below this threshold (*P*<0.001 for all; Figure S2A through S2C). Gradient of risk of MR-proADM levels for predicting all-cause death and the composite end point was evaluated using fractional polynomial regression. In univariable assessment, MR-proADM was statistically associated with mortality and the composite end point (*P*<0.001). After multivariable adjustment with NT-proBNP and eGFR (model 1) and with NT-proBNP, eGFR, NYHA and diuretic dose (model 2), MR-proADM persisted to be significantly associated with all-cause mortality and the composite end point in both models (Figure 1).

#### Optimal Cutoff of MR-proADM for ATTR-CM

**Figure 2.**
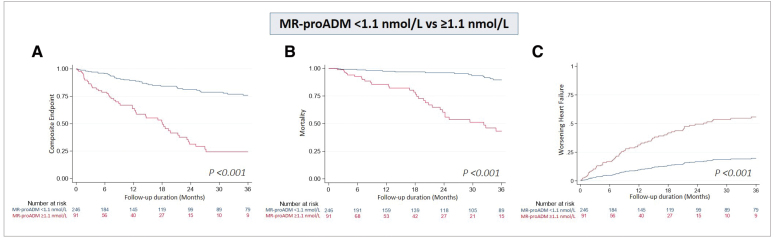
**Kaplan-Meier curves for freedom from the composite end point (A), all-cause mortality (B), and cumulative incidence of worsening heart failure events (C).** All stratified by MR-proADM levels (<1.1 nmol/L in blue vs ≥1.1 nmol/L in red). All *P*<0.001. MR-proADM indicates mid-regional pro-adrenomedullin.

The optimal cutoff value of MR-proADM at 1 year in our cohort was 1.1 nmol/L. Patients with MR-proADM levels above this threshold experienced worse outcomes (*P*<0.001 for all end points; Figure 2). Median survival among patients with MR-proADM <1.1 nmol/L was not reached (95% CI, 74.5 months; could not be estimated), compared with 29.4 months (95% CI, 21.2–37.5) in those with MR-proADM ≥1.1 nmol/L. Among patients with MR-proADM <1.1 nmol/L, 1-year and 3-year survival rates were 96.9% (93.2–98.6%) and 87.6% (80.4–92.2%), respectively, compared with 82.8% (72.1–89.7%) and 40.5% (26.9–53.8%) in those with MR-proADM ≥1.1 nmol/L (*P*<0.001).

Overall, 91 patients (27%) had MR-proADM levels >1.1 nmol/L. Baseline characteristics stratified by this threshold are summarized in Table S2.

When stratifying by site, MR-proADM remained strongly associated with mortality in patients from the Hospital Puerta de Hierro (*P*<0.001), whereas in those from the Hospital Germans Trias i Pujol, the association did not reach statistical significance despite the direction of the effect was consistent, likely explained by the low number of patients included from this center (Figure S3).

#### Additive Value to Current Staging Systems

**Figure 3.**
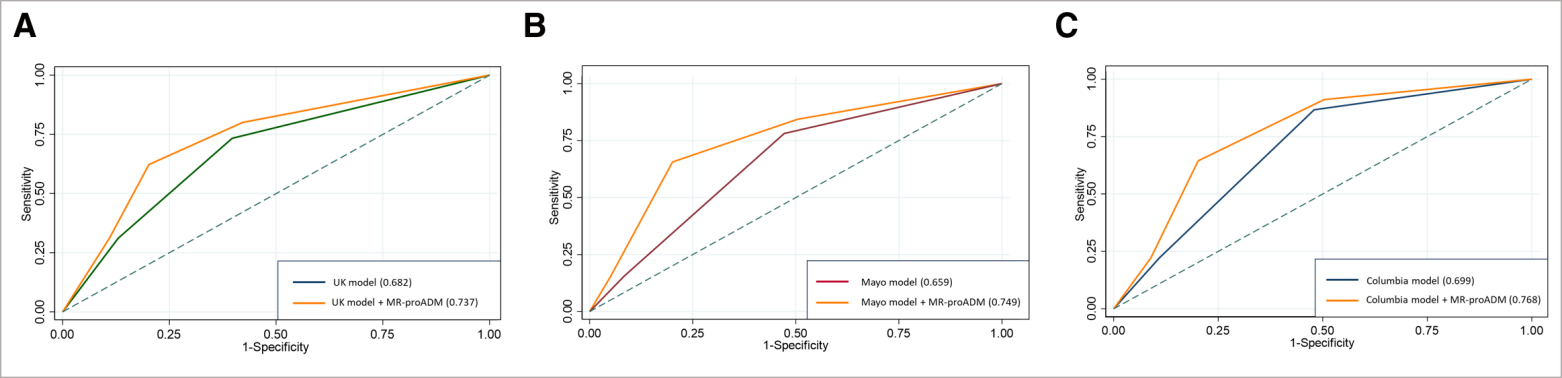
**ROCs displaying the incremental benefit of adding MR-proADM to the NAC staging system (A), modified Mayo staging system (B), and to the Columbia staging system (C) for predicting all-cause mortality.** MR-proADM values were stratified into <1.1 nmol/L and ≥1.1 nmol/L. MR-proADM indicates mid-regional pro-adrenomedullin; NAC, National Amyloid Center; and ROC, receiver-operating curve.

In our cohort, the NAC staging system achieved an AUC of 0.682 (95% CI, 0.604–0.760) for predicting 3-year mortality. The addition of MR-proADM, dichotomized with a cutoff of 1.1 nmol/L, enhanced the AUC to 0.737 (95% CI, 0.661–0.813; Figure 3A). Additionally, Harrell’s C-index significantly improved with the inclusion of MR-proADM from 0.694 (95% CI, 0.624–0.765) for the NAC model alone to 0.765 (95% CI, 0.691–0.838) when combined with MR-proADM, with a *P* value for the difference of 0.005 (Table 4).

**Table 4.**
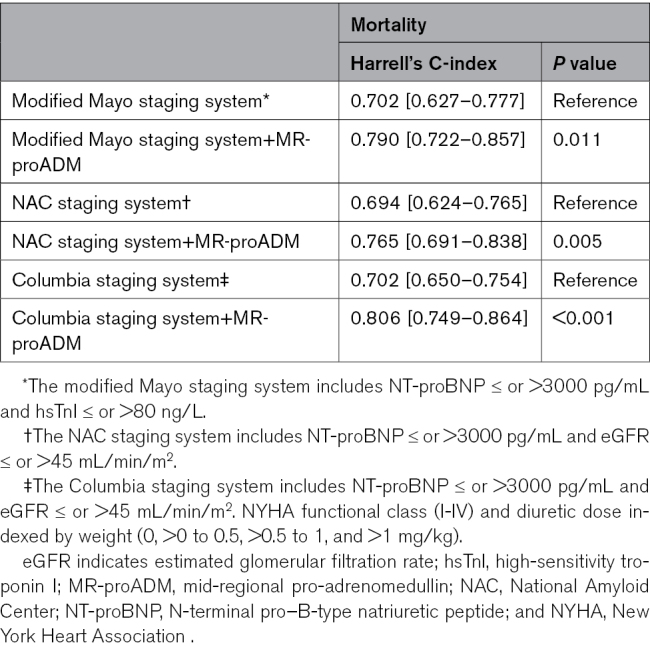
Incremental Benefit of Adding MR-proADM to the Modified Mayo, NAC, and the Columbia Staging Systems for Predicting All-Cause Mortality

The modified Mayo staging system yielded an AUC of 0.659 (95% CI, 0.572–0.746), improving to 0.749 (95% CI, 0.659–0.83) after addition of MR-proADM (Figure 3B). Similarly, Harrell’s C-index increased from 0.702 (95% CI, 0.627–0.777) to 0.790 (95% CI, 0.722–0.857; *P*=0.011; Table 4).

Finally, the Columbia staging system achieved an AUC of 0.699 (95% CI, 0.632–0.766), and incorporation of MR-proADM improved the AUC to 0.768 (95% CI, 0.702–0.833; Figure 3B). This improvement was also reflected in Harrell’s C-index, which increased from 0.702 (95% CI, 0.650–0.754) to 0.806 (95% CI, 0.749–0.864), with a *P* value for the difference <0.001 (Table 4).

#### External Validation

The first external validation cohort consisted of 210 US patients with ATTR-CM (median age of 77.8 years [73.55–82.26 years]), of whom 90.5% were men, 81.9% had ATTRwt, and 60% were on tafamidis treatment (Table S3). Patients in this cohort exhibited a more advanced NYHA functional class and had been previously admitted for heart failure more frequently than in the derivation cohort. Patients showed slightly lower eGFR and LVEF values and were treated with tafamidis more frequently (60% versus 25.5%; *P*<0.001), reflecting the earlier availability of tafamidis in the United States compared with Spain. All other baseline characteristics were consistent between the 2 cohorts.

**Figure 4.**
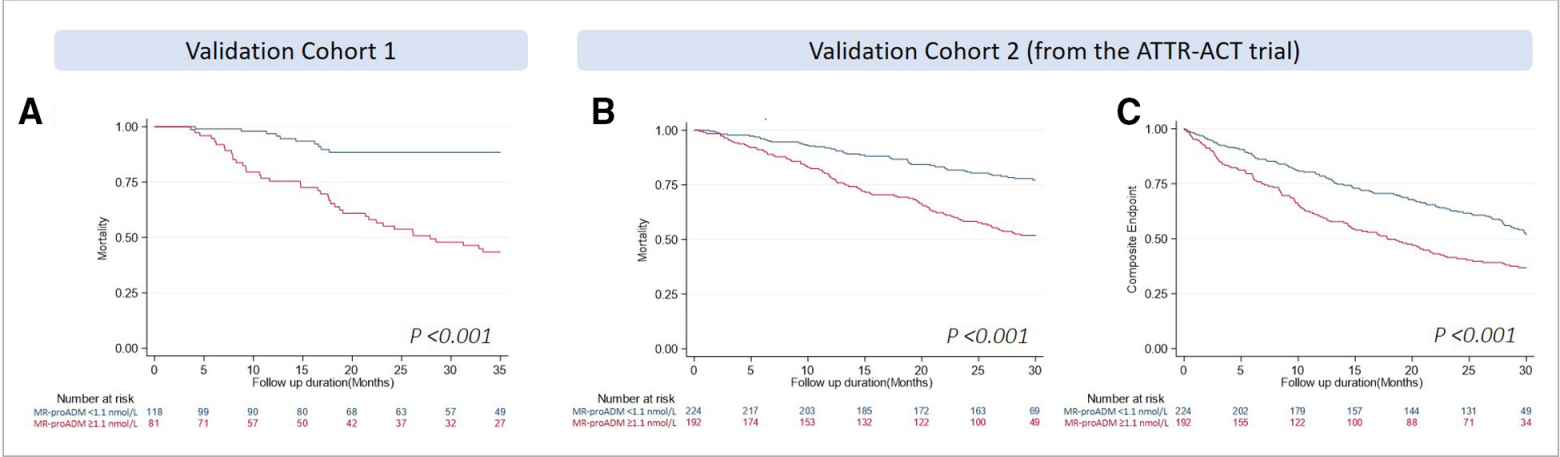
**Kaplan-Meier curves for all-cause mortality in validation cohort 1 (A) and for all-cause mortality and the composite end point in validation cohort 2 (B and C).** All *P*<0.001.

Over a median follow-up of 22.8 months (IQR, 7.9–48.4), there were 70 deaths (33.3%) and 5 heart transplants (2.4%). Consistent with the derivation cohort, MR-proADM demonstrated a good predictive performance for the end point of all-cause mortality, with a Harrell’s C-index of 0.725 (95% CI, 0.646–0.805). Patients with MR-proADM <1.1 nmol/L did not reach median survival, with a 1-year survival of 97% (91–99%) and 3-year survival of 88.5% (79.6–93.6%). In contrast, patients with MR-proADM ≥1.1 nmol/L had a median survival of 27 months (95% CI, 19.1; could not be estimated), with a 1-year survival of 75% (64–84%) and a 3-year survival of 39.2% (27.9–50.2%; Figure 4A).

These findings were subsequently validated in a second validation cohort of 416 patients from the ATTR-ACT trial. Participants exhibited a median age of 75 years (71–79); 90.6% were men, 76.9% had wild-type ATTR, and 59.9% were receiving tafamidis (Table S3). Compared with the derivation cohort, this group had a more advanced NYHA class, higher NT-proBNP levels, lower eGFR and LVEF, and a greater proportion of patients were receiving diuretic therapy. These characteristics likely reflect a more advanced disease stage and are consistent with the earlier recruitment period of this cohort (2013 to 2015).

Over a follow-up period of 30 months, 136 patients died (32.7%), 171 (41.1%) were hospitalized for heart failure, 11 (2.6%) underwent heart transplantation, and 3 (0.72%) received a left ventricular assist device.

Patients with MR-proADM levels ≥1.1 nmol/L exhibited a significantly higher incidence of mortality and the composite outcome of mortality and heart failure hospitalization (Figure 4B and 4C). Findings remained consistent in the subgroup of patients receiving tafamidis (Supplemental Figures S4A and S4B).

## Discussion

This study is the first to systematically evaluate multiple circulating biomarkers related to heart failure in ATTR-CM. Our findings highlight the potential of using MR-proADM to assess prognosis in ATTR-CM, a condition with a unique pathophysiology that differs from other forms of heart failure. Our work shows that increased MR-proADM levels are associated with several markers of severity of the disease and are independently associated with all-cause death and heart failure events. Moreover, we identified a specific cutoff value for MR-proADM that accurately identified patients with poorer outcomes independently of currently established prognostic biomarkers like NT-proBNP and eGFR. Lastly, we confirmed the incremental value of MR-proADM when added to the NAC, Mayo, and Columbia prognostic staging systems and validated the prognostic capacity of the proposed MR-proADM cutoff in 2 external cohorts.

The landscape of amyloidosis has undergone outstanding changes in recent years.^1,2^ With the advances in noninvasive diagnosis and the development of specific treatments, a growing number of patients are being diagnosed at earlier stages of the disease, and the prognosis has significantly improved.^22,23^ Consequently, there is a pressing need for tools to accurately stratify the risk of these patients, identifying those requiring closer monitoring and those likely to benefit the most from targeted treatments including the possibility of aggressive approaches combining several specific therapies.

The first significant contribution to predict prognosis in ATTR-CM was made in 2016 by Grogan et al, who demonstrated that NT-proBNP and troponin T were independent predictors of mortality in ATTRwt, and created the first staging system based on the values of both biomarkers.^8^ In 2018, Gillmore et al proposed and validated the NAC staging system, based on NT-proBNP and eGFR levels, that predicted survival in both ATTRwt and ATTRv patients.^7^ This staging system has since gained recognition and is widely used in clinical practice and clinical trials. Building upon this foundation, Cheng et al identified weight-indexed diuretic dose and NYHA functional class as additional independent predictors of mortality that provided incremental value to previous scoring systems.^13^ Our study aims to make further progress in this field, considering that patient profiles are evolving, specific therapies are now routinely prescribed, and survival is increasing.

Moreover, whereas mortality remains the main concern of physicians treating ATTR-CM patients, short-term challenges of patients with ATTR-CM often revolve around fluid management and heart failure decompensations. Hospitalizations attributable to heart failure have been shown to worsen prognosis in ATTR-CM^9^ and are strongly related with a decrease in quality of life,^24^ underscoring the need to accurately identify those individuals at risk to closely monitor and adjust their diuretic therapy to prevent worsening heart failure episodes and improve prognosis. In fact, a recent study has demonstrated that NT-proBNP progression and outpatient diuretic intensification at 1 year are associated with an increased risk of subsequent mortality in ATTR-CM.^25^

Adrenomedullin (ADM) is a 52-amino acid peptide belonging to the calcitonin gene-related peptide family. It is present in nearly all human tissues, with the highest concentrations being found in the adrenal medulla, heart, lungs, and endothelium.^20^

In the setting of heart failure, the ADM gene is upregulated in cardiac myocytes because of pressure and volume overload. ADM acts as a vasodilator and stimulates diuresis and natriuresis, effectively reducing both preload and afterload.^26^ Moreover, ADM plays a role in reducing cardiac remodeling and fibrosis.^27^ It also stabilizes the endothelial barrier, modulates its permeability, and exerts an anti-inflammatory effect. ADM could be of particular interest in amyloidosis, as the disease has shown frequent vascular involvement, with perivascular deposits and capillary infiltration.^28^ In fact, elevated ADM levels have been described not only in heart failure but also in other conditions in which permeability of vessels could be affected such as sepsis, COVID-19, and inflammatory diseases.^29^

However, determination of ADM concentrations is limited because of its in vitro instability, attributed to its short half-life and rapid clearance from the bloodstream.^30^ This problem was solved by measuring MR-proADM, a stable fragment of pro-ADM, in which concentrations accurately reflect those of ADM.^31,32^

Adlbrecht et al demonstrated that the prognostic value of MR-proADM was higher than that of BNP in a cohort of 786 patients with chronic heart failure.^33^ Furthermore, they observed that MR-proADM was a better predictor of mortality in patients with nonischemic heart failure. Later, Von Haehling et al confirmed that MR-proADM was an independent predictor of 12-month mortality in a group of 501 chronic heart failure patients with MR-proADM adding prognostic value to NT-proBNP.^20^

In the present study, we demonstrate that MR-proADM behaves as an independent predictor for mortality and for the composite end point of all-cause death, worsening heart failure and heart transplant in patients with ATTR-CM. Although MR-proADM has not been previously investigated in amyloidosis, our findings are consistent with the scarce evidence available in heart failure. In the BACH trial (Biomarkers in Acute Heart Failure), Maisel et al demonstrated that for patients presenting to the emergency department with dyspnea, MR-proADM identified those at higher risk of 90-day mortality, and that MR-proADM added prognostic value to BNP.^34^ In the VERifying DYspnea trial, Travaglino et al further confirmed its prognostic value, demonstrating that MR-proADM was a strong predictor of 90-day mortality in patients admitted for dyspnea.^35^ Consistent with its prognostic role, our study demonstrated the association between increased MR-proADM levels and the presence of multiple indicators of severity in ATTR-CM.

To our knowledge, this is the first study to evaluate MR-proADM in ATTR-CM. Our findings highlight the promising potential of this biomarker in this clinical scenario. This is particularly important considering the increasing life expectancy of patients with ATTR-CM and the imperative need to identify those at higher risk of heart failure decompensation, who need closer monitoring and tailored diuretic treatment. We identified a threshold (MR-proADM ≥1.1 nmol/L) that predicts worse prognosis and validated it in 2 independent cohorts: a real-world cohort, reflecting everyday clinical practice, and a clinical trial cohort from the ATTR-ACT study. In both settings, MR-proADM was consistently associated with the incidence of adverse cardiovascular outcomes. Furthermore, MR-proADM could be used to improve patient selection in new clinical trials, decreasing the number of patients needed to demonstrate clinical benefit in a scenario of patients already receiving specific therapies. Lastly, our findings open avenues for further research and bring the opportunity to evaluate changes in this biomarker as short-term indicators of deterioration that could assist clinicians when switching or adding specific therapies.

### Study Limitations

In the derivation cohort, the number of patients who received tafamidis was low, and both women and patients with ATTRv were underrepresented. Heart failure events were not available in one of the validation cohorts. MR-proADM is not a biomarker commonly used in clinical practice. However, several MR-proADM assays are available, which may allow rapid adoption in routine laboratory testing. Because this biomarker is not specific to heart disease, its applicability may be limited in the presence of other conditions such as sepsis or COVID-19 infection, which warrants further research.

### Conclusions

In the present study, we demonstrate that MR-proADM is an independent predictor of all-cause mortality and the composite end point of all-cause mortality, worsening heart failure and heart transplant in patients with ATTR-CM. MR-proADM improved prediction when added to the Mayo, NAC, and Columbia staging systems. Our work paves the way for tailored risk assessment and targeted management strategies in ATTR-CM.

## Article Information

### Disclosures

M.M. reports grants from Alnylam, BridgeBio, Intellia, and Ionis, and personal fees from Alnylam, Novo-Nordisk, Roche, Prothena Astra Zeneca, Akcea, and Intellia. A.M. reports research grants from Pfizer, Ionis, Attralus, and Cytokinetics, and fees from Cytokinetics, BMS, Eidos/BridgeBio, Pfizer, Ionis, Lexicon, Attralus, Alnylam, Haya, Alexion, Akros, Prothena, BioMarin, AstraZeneca, and Tenaya. The other authors have nothing to disclose.

### Supplemental Material

Supplemental Methods

Tables S1–3

Figures S1–S4

## Supplementary Material
