# Supplementary material for: MR-proADM Predicts Mortality and Heart Failure Events in ATTR Cardiac Amyloidosis
Source: Circulation. 2026 Mar 31;153(18):1350–61. doi: 10.1161/CIRCULATIONAHA.125.077833 (PMC13127803; doi:10.1161/CIRCULATIONAHA.125.077833)

SUPPLEMENTAL MATERIAL

Peiró-Aventín et al.

MR-proADM predicts mortality and heart failure events in ATTR cardiac amyloidosis

1. **Supplemental methods: Assays and laboratory analysis.**
2. **Supplemental Tables.**
3. **Supplemental Figures.**

1. **Supplemental methods: Assays and laboratory analysis.**

Supplemental methods: Assays and laboratory analysis

CA125, Galectin-3, CD146, α-Klotho, sST2, MR-proADM, FGF-23, NT-proBNP and HsTnI were determined from plasma, while IGFBP-7 and GDF-15 were assessed in serum. All samples were collected under standardized conditions.

CA125

Human CA125 was measured with the ARCHITECT CA 125 II assay, a chemiluminescent microparticle immunoassay (CMIA) on the ARCHITECT *i* System (Abbott Laboratories, ref. 2K45; derivation cohort lot 44135FP00; validation cohort: lot 52557FP00). The ARCHITECT CA 125 II assay precision was ≤10% total CV and analytical sensitivity and specificity were ≤10 U/mL and ≤12%, respectively.

Galectin-3

Human Galectin-3 levels were measured with the ARCHITECT Galectin-3 assay, a chemiluminescent microparticle immunoassay (CMIA) on the ARCHITECT *i* System (Abbott Laboratories, ref. 5P03; lot 44036FP00). The ARCHITECT Galectin-3 assay precision was ≤10% total CV and the limit of detection (LOD) and quantitation (LOQ) were 1.1 ng/mL and ≤4 ng/mL, respectively.

CD146

Soluble CD146 was measured using the CY-QUANT ELISA sCD146 (BioCytex; ref. 7501; lot. 220452). Samples were diluted 1:10 in dilution buffer (reagent 4). Measurement range was 10 – 160 ng/mL. The method of analysis was according to the manual provided by the supplier.

Alpha-Klotho

Soluble alpha-klotho levels were quantitative determined by Soluble Alpha-Klotho ELISA Kit (IBL international; ref. JP27998; lot 2B-302). Samples were diluted 1:2 in EIA Buffer. Measurement range and sensitivity were 93.75 - 6000 pg/mL and 6.15 pg/mL, respectively. The method of analysis was according to the manual provided by the supplier.

sST2

Soluble ST2 was analyzed with the ST2 Presage (Critical Diagnostics; ref. #BC-1065E; lot 228K063). Samples were diluted 1:50 in sample diluent. The LOD and LOQ are 1.8 ng/mL and 2.4 ng/mL, respectively. The method of analysis was according to the manual provided by the supplier.

MRproADM

Human MRproADM concentrations were analyzed with the LIAISON® BRAHMS MRproADM assay, an automated chemiluminescent immunoassay (CLIA) on the LIAISON® system (DiaSorin Inc, ref. 318480; derivation and validation cohort lot 136655). The LIAISON® BRAHMS MRproADM assay measures between 0.21 nmol/L and 10nmol/L.

FGF23

Human FGF23 was analyzed with the LIAISON® FGF23 assay, an automated chemiluminescent immunoassay (CLIA) on the LIAISON® system (DiaSorin Inc, ref. 318700; lot 247013). The LOD and LOQ are 5 pg/mL and 6.5 pg/mL, respectively.

NT-proBNP

NT-proBNP concentrations were measured with the Elecsys proBNP II STAT (Roche Diagnostics, Indianapolis, Indiana; code No. 05390109), an immunoelectrochemiluminescence assay (ECLIA) on a Modular Analytics Cobas e 601 analyzer (Roche Diagnostics, Indianapolis, Indiana). The sensitivity of the assay was 5.9 pmol/L. The measurement interval was 0.6-4130 pmol/L (defined by the limit of detection and the maximum of the master curve).

HsTnI

High-sensitivity cardiac troponin I (hs-cTnI) was measured with the Atellica® IM High Sensitivity Troponin I assay (Siemens Healthineers, ref. 10997841), a chemiluminescent sandwich immunoassay on the Atellica® IM Analyzer. The assay has a total coefficient of variation (CV) ≤10% at the 99th percentile upper reference limit, with a limit of detection of approximately 1.6 ng/L and an analytical range up to 25,000 ng/L.

GDF-15

GDF-15 was determined by the Human GDF-15 Quantikine ELISA Kit (R&D Systems, ref. SGD150; lot P347781) following the guidelines of the manufacturer. Samples were diluted 1:4 in Calibrator Diluent RD5-20. The minimum detectable dose was 23.4 pg/mL. The analysis was performed in the Bio-Rad Benchmark Plus microplate spectrophotometer and used the software Bio-Rad Microplate Manager version 5.2.1 build 106.

IGFBP-7

IGFBP7 levels were measured using the Human IGFBP7 ELISA Kit (Abcam, ref. ab213790; lot GR3434975-1) following the guidelines of the manufacturer. Samples were diluted 1:10 in diluent buffer. The biological sensitivity of the assay was <20 pg/mL. The range was 625 – 40000 pg/mL. The analysis was performed in the Bio-Rad Benchmark Plus microplate spectrophotometer and used the software Bio-Rad Microplate Manager version 5.2.1 build 106.

2. **Supplemental tables.**

Table S1. Baseline Characteristics Stratified by Site in the Derivation Cohort.

	Overall cohort<br><br>N=337	Hospital Universitario Puerta de Hierro (Madrid)<br><br>N= 296	Hospital Germans Trias I Pujol (Barcelona)<br><br>N=41	*p* value
**Age (years), median [IQR]**	78.27 [73.09 – 82.92]	78.02 [72.79 – 82.4]	82.4 [77.38 – 85.95]	0.002
**Male gender**	276 (81.9%)	247 (83.45%)	29 (70.73%)	0.048
**ATTR type**				0.026
**Wild-type**	291 (86.35%)	251 (84.8%)	40 (97.56%)	
**Hereditary**	46 (13.65%)	45 (15.2%)	1 (2.44%)	
**Systolic blood pressure (mmHg), median [IQR]**	130 [119 – 140]	130 [119 – 140]	126 [116 – 136]	0.555
**NYHA functional class**				0.004
**I**	92 (27.3%)	89 (30.07%)	3 (7.32%)	
**II**	182 (54.01%)	151 (51.01%)	31 (75.61%)	
**III**	63 (18.69%)	56 (18.92%)	7 (17.07%)	
**History of atrial fibrillation/flutter**	206 (61.13%)	177 (59.79%)	29 (70.73%)	0.186
**Previous heart failure admissions**	125 (37.09%)	97 (32.77%)	28 (68.29%)	<0.001
**Stroke**	28 (8.31%)	21 (7.09%)	7 (17.07%)	0.03
**Ischemic cardiomyopathy**	53 (15.73%)	45 (15.2%)	8 (19.51%)	0.477
**NTproBNP (pg/mL), median [IQR]**	2209 [942 – 4140]	1999 [859 – 3933]	3020 [1655 – 6807]	0.005
**eGFR (mL/min), median [IQR]**	61 [47 – 78]	62 [47 – 78]	59 [42 – 80]	0.37
**LVEF (%), median [IQR]**	55 [46 – 60]	54 [46.6 – 60]	55 [45 – 61]	0.933
**LVEDD (mm), median [IQR]**	44 [39 – 47]	43 [39 – 47]	48 [43 – 53]	<0.001
**LA diameter (mm), median [IQR]**	45 [41 – 49]	44 [41 – 49]	47 [45 – 53]	<0.001
**Lateral E/e' , median [IQR]**	13.5 [10.6 – 18.6]	13.5 [10.4 – 18.7]	15.1 [12 – 18]	0.253
**TAPSE (mm), median [IQR]**	18 [15 – 21]	18 [15 – 21]	17 [15 – 20]	0.093
**Pericardial effusion**	56 (16.62%)	47 (15.9%)	9 (21.95%)	0.275
**GLS (%), median [IQR]**	-12.9 [-15.1 to -9.7]	- 12.9 [-15.1 to -9.7]	__	0.431
**PASP (mmHg), median [IQR]**	45 [40 – 52]	45 [40 – 53]	45 [39 – 52]	0.846
**Treatment**				
**Betablockers**	133 (39.47%)	115 (38.85%)	18 (43.9%)	0.420
**Diuretics**	228 (67.66%)	197 (66.55%)	31 (75.61%)	0.245
**Furosemide dose (mg/kg), median [IQR]**	0.47 [0 – 0.87]	0.44 [0 – 0.90]	0.55 [0.11 – 0.76]	0.668
**Tafamidis (at baseline or initiated during the initial 12 months of follow-up)**	92 (27.3%)	90 (30.4%)	2 (4.88%)	0.001

Table S2. Association of MR-proADM (cut-off 1.1 nmol/L) with severity of the disease.

	MR-proADM		
	**< 1.1 nmol/L N= 246**	**≥ 1.1 nmol/L N= 91**	**p**
Age (years), median [IQR]	77.6 [71.76 – 81.21]	82 [76.75 – 86.02]	<0.001
Male gender	205 (83.33%)	71 (78.02%)	0.261
ATTR type			0.008
Wild-type	205 (83.33%)	86 (94.51%)	
Hereditary	41 (16.67%)	5 (5.49%)	
Systolic blood pressure (mmHg), median [IQR]	132 [120 – 143]	125 [114 – 133]	0.004
NYHA functional class			<0.001
I	88 (35.77%)	4 (4.4%)	
II	133 (54.07%)	49 (53.85%)	
III	25 (10.16%)	38 (41.75%)	
History of atrial fibrillation/flutter	129 (52.65%)	77 (84.62%)	<0.001
Previous heart failure admission	71 (28.86%)	54 (59.34%)	<0.001
Stroke	20 (8.13%)	8 (8.79%)	0.845
Ischemic cardiomyopathy	32 (13.01%)	21 (23.08%)	0.024
NT-proBNP (pg/mL), median [IQR]	1522 [750 – 2705]	5518 [3403 – 9052]	<0.001
eGFR (mL/min), median [IQR]	68 [55 – 82]	42 [33 – 50]	<0.001
hsTnI (ng/L), median [IQR]	42 [29.1 – 71]	54 [29.4 – 129.9]	0.080
LVEF (%), median [IQR]	56 [47 – 60]	50 [43 – 60]	0.009
LVEDD (mm), median [IQR]	44 [40 – 47]	43 [39 – 47]	0.808
LA diameter (mm), median [IQR]	45 [41 – 49]	47 [43 – 49]	0.026
Lateral E/e', median [IQR]	13.4 [10.4 – 18]	15.1 [10.9 – 19.3]	0.086
TAPSE (mm), median [IQR]	19 [16 – 22]	17 [13 – 19]	<0.001
Pericardial effusion	37 (15.04%)	19 (20.88%)	0.137
GLS (%), median [IQR]	-13.3 [-15.9 to -10.3]	-10.5 [-13.1 to -7.7]	<0.001
PASP (mmHg), median [IQR]	43 [36 – 50]	50 [40 – 60]	<0.001
**Treatment**			
Betablockers	83 (33.74%)	50 (54.95%)	<0.001
Diuretics	142 (57.72%)	86 (94.51%)	<0.001
Furosemide dose (mg/kg), median [IQR]	0.24 [0 – 0.65]	0.86 [0.55 – 1.21]	<0.001
Tafamidis (Baseline or initiated during the initial 12 months of follow-up)	71 (28.86%)	21 (23.08%)	0.281
**Outcomes**			
All-cause death	28 (11.38%)	38 (41.76%)	<0.001
Worsening HF	55 (22.36%)	44 (48.35%)	<0.001
Composite endpoint*	53 (21.54%)	51 (56.04%)	<0.001

* All-cause death, worsening heart failure and heart transplant.

ATTR: transthyretin amyloidosis; eGFR: estimated glomerular filtration rate; GLS: global longitudinal strain; HF: heart failure; hsTnI: high sensitivity troponin I; LA: left atrium; LVEDD: left ventricular end-diastolic diameter; LVEF: left ventricular ejection fraction; NYHA: New York Heart Association; PASP: pulmonary artery systolic pressure; TAPSE: tricuspid annular plane systolic excursion.

Table S3. Characteristics of the Validation Cohorts.

	DERIVATION COHORT (Spain) N=337	VALIDATION COHORT 1 (United States) N=210	p*	VALIDATION COHORT 2 (ATTR-ACT) N=416	p†
**Age (years), median [IQR]**	78.27 [73.09 – 82.92]	77.81 [73.55 – 82.26]	0.695	75.00 [71.00 – 79.00]	<0.001
**Male gender**	276 (81.9%)	190 (90.48%)	0.006	377 (90.63%)	<0.001
**ATTR type**			0.161		0.001
**Wild-type**	291 (86.35%)	172 (81.9%)		320 (76.92%)	
**Hereditary**	46 (13.65%)	38 (18.1%)		96 (23.10%)	
**NYHA functional class**			<0.001		<0.001
**I**	94 (27.89%)	26 (12.38%)		36 (8.65%)	
**II**	181 (53.71%)	90 (42.86%)		251 (60.34%)	
**III**	62 (18.4%)	93 (44.29%)		129 (31.01%)	
**IV**	-	1 (0.48%)		-	
**History of atrial fibrillation/flutter**	204 (60.71%)	139 (66.19%)	0.198	252 (60.58%)	0.969
**Ischemic cardiomyopathy**	52 (15.43%)	68 (32.38%)	<0.001	11 (2.64%)	<0.001
**NT-proBNP (pg/mL), median [IQR]**	2209 [906 – 4100]	2186 [951 – 3633]	0.675	3024 [1784-4778]	<0.001
**eGFR (mL/min), median [IQR]**	62 [47 – 79]	56 [41 – 70]	0.011	54 [46 – 65]	<0.001
**LVEF (%), median [IQR]**	54.5 [46 – 60]	52.5 [43 – 58]	0.01	49.4 [41.4 – 55.6]	<0.001
**Diuretics**	222 (65.88%)	154 (73.33%)	0.067	371 (89.18%)	<0.001
**Furosemide dose, mg/kg**	0.44 [0 – 0.86]	0.3 [0.07 – 0.63]	0.79	not available	-
**Tafamidis (at baseline or initiated during the initial 12 months of follow-up)**	86 (25.52%)	126 (60%)	<0.001	249 (59.86%)	<0.001

ATTR: transthyretin amyloidosis; eGFR: estimated glomerular filtration rate; LVEF: left ventricular ejection fraction; NYHA: New York Heart Association.

*p-value for comparison between derivation cohort and validation cohort 1.

†p-value for comparison between derivation cohort and validation cohort 2.

3. Supplemental Figures

Figure S1. Kaplan-Meier Curves for Freedom from the Composite Endpoint (S1A), All-Cause Mortality (S1B) and Cumulative Incidence of Worsening Heart Failure Events (S1C) in the Derivation Cohort.

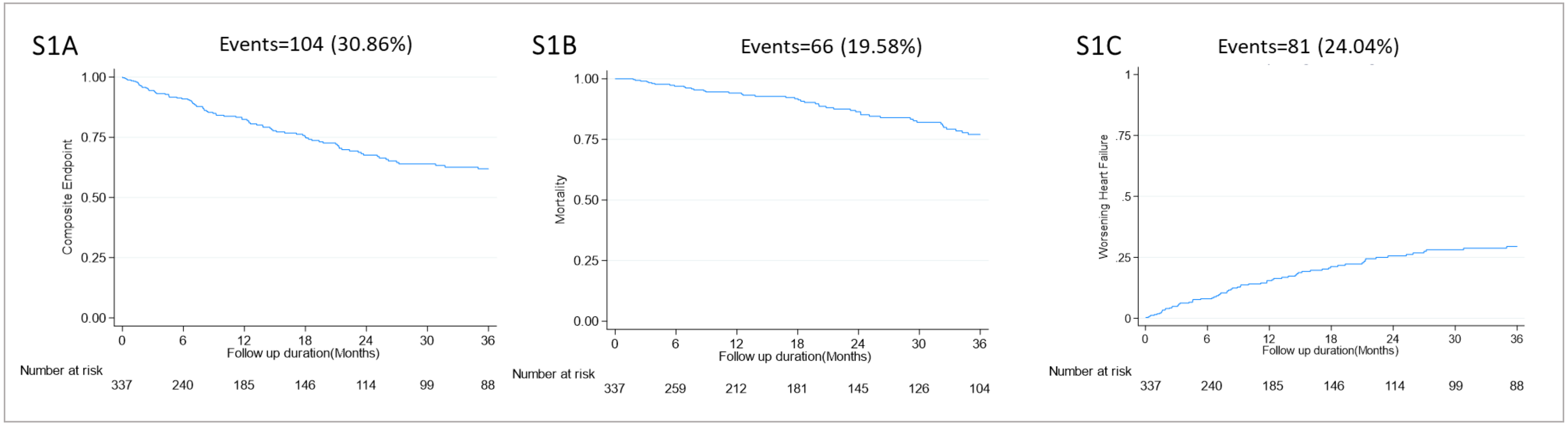

Figure S2. Kaplan–Meier curves for Freedom from the Composite Endpoint (S2A), All-Cause Mortality (S2B) and Cumulative Incidence of Worsening Heart Failure Events (S2C) by MR-proADM levels: < 0.79 nmol/L (blue) vs ≥ 0.79 nmol/L (red). (All p < 0.001).

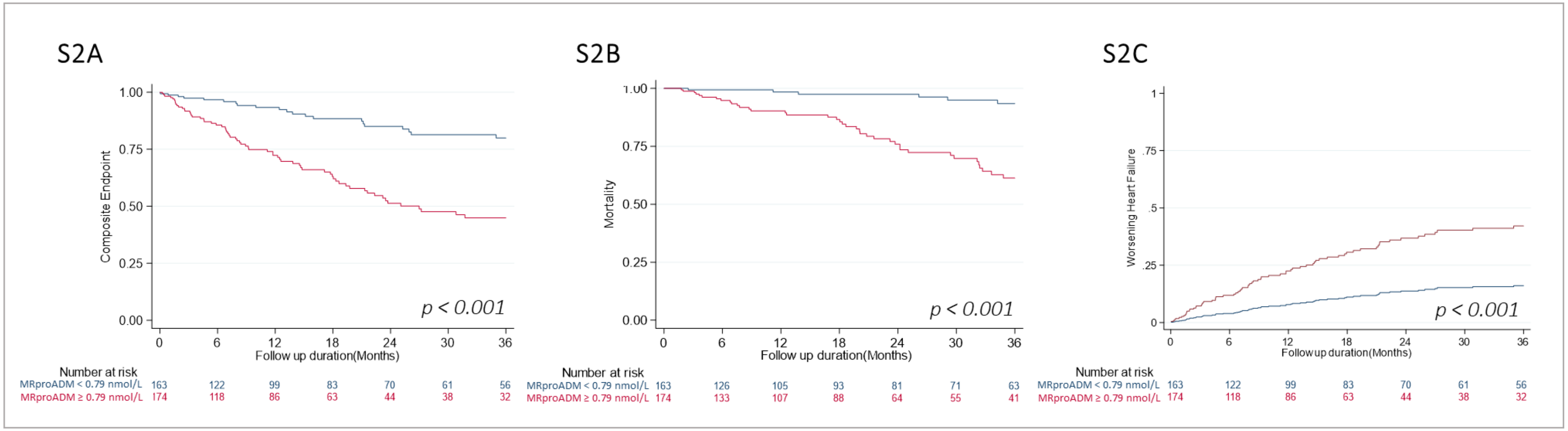

Figure S3. Kaplan–Meier Curves for Freedom from All-Cause Mortality Stratified by Site in the Derivation Cohort.

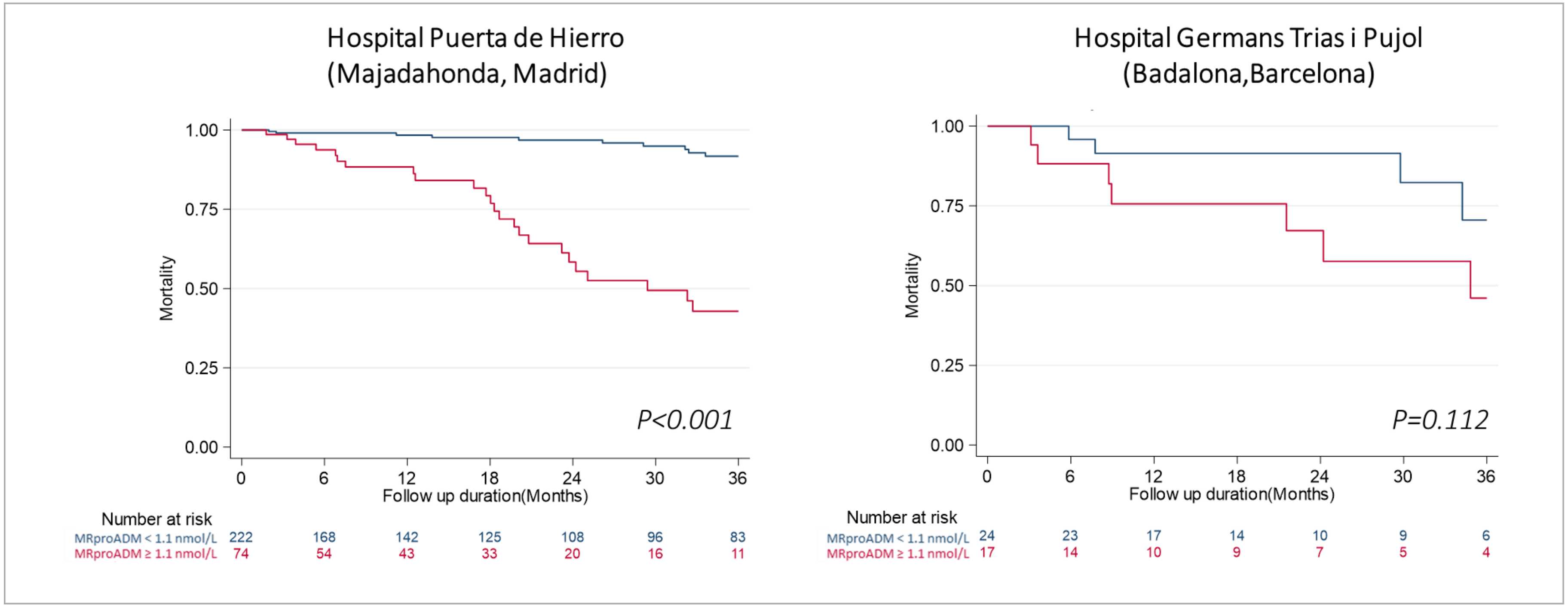

Figure S4. Kaplan–Meier curves for All-Cause Mortality (S4A) and the Composite Endpoint (S4B) in Patients Treated with Tafamidis in the ATTR-ACT trial (Validation Cohort 2) by MR-proADM levels: < 1.1 nmol/L (blue) vs ≥ 1.1 nmol/L (red).

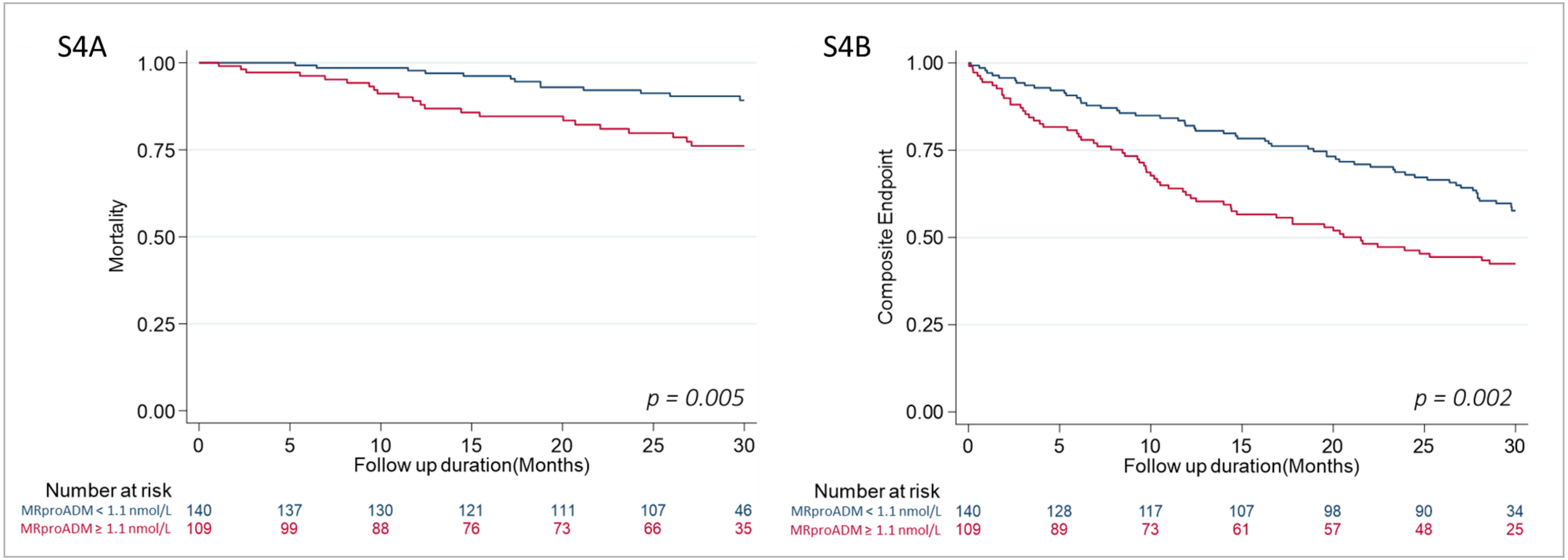

Supplement: Supplementary file 1 [file cir-153-1350-s001.pdf]
